# Stereotactic anatomy of the third ventricle

**DOI:** 10.1007/s00276-024-03312-1

**Published:** 2024-02-19

**Authors:** Alexandra Campos da Silva, Susana Maria Silva, Hélio Alves, Diogo Cunha-Cabral, Filipe F. Pinto, João Fernandes‑Silva, Mavilde Arantes, José Paulo Andrade

**Affiliations:** 1 Unit of Anatomy, Department of Biomedicine, Faculty of Medicine, University of Porto, Alameda Professor Hernâni Monteiro, 4200‑319 Porto, Portugal; 2 CINTESIS@RISE, Rua Dr. Plácido da Costa, s/n, 4200‑450 Porto, Portugal; 3 Division of Neuroradiology, Radiology Service, Portuguese Institute of Oncology, Rua Dr. António Bernardino de Almeida 865, 4200‑072 Porto, Portugal; 4 Health Local Unit of Matosinhos Otorhinolaryngology, Hospital Pedro Hispano, Rua Dr. Eduardo Torres, 4464-513, Matosinhos, Portugal

**Keywords:** Third ventricle, Neuroanatomy, Endoscopic third ventriculostomy, Neuronavigation, Stereotactic coordinates, Hydrocephalus

## Abstract

**Purpose:**

Endoscopic third ventriculostomy (ETV) is a surgical procedure that can lead to complications and requires detailed preoperative planning. This study aimed to provide a more accurate understanding of the anatomy of the third ventricle and the location of important structures to improve the safety and success of ETV.

**Methods:**

We measured the stereotactic coordinates of six points of interest relative to a predefined stereotactic reference point in 23 cadaver brain hemi-sections, 200 normal brain magnetic resonance imaging (MRI) scans, and 24 hydrocephalic brain MRI scans. The measurements were statistically analyzed, and comparisons were made.

**Results:**

We found some statistically significant differences between genders in MRIs from healthy subjects. We also found statistically significant differences between MRIs from healthy subjects and both cadaver brains and MRIs with hydrocephalus, though their magnitude is very small and not clinically relevant. Some stereotactic points were more posteriorly and inferiorly located in cadaver brains, particularly the infundibular recess and the basilar artery. It was found that all stereotactic points studied were more posteriorly located in brains with hydrocephalus.

**Conclusion:**

The study describes periventricular structures in cadaver brains and MRI scans from healthy and hydrocephalic subjects, which can guide neurosurgeons in planning surgical approaches to the third ventricle. Overall, the study contributes to understanding ETV and provides insights for improving its safety and efficacy. The findings also support that practicing on cadaveric brains can still provide valuable information and is valid for study and training of neurosurgeons unfamiliar with the ETV technique.

## Introduction

Shunting procedures for cerebrospinal fluid (CSF) diversion have been used for years to treat hydrocephalus [1]. Endoscopic third ventriculostomy (ETV) is now a viable alternative to shunting procedures and consists in opening a burr hole 0.5 cm anterior to the coronal suture and 2.5 cm lateral to the midline, inserting an endoscope that travels through the lateral ventricle and interventricular foramen (of Monro) (IVF) [1]. The anatomic landmarks of the lateral ventricle, i.e., the septal and thalamostriate veins and the choroid plexus, must be clearly visualized [2–4]. After reaching the third ventricle floor, the latter is perforated between the rostral infundibular recess and mammillary bodies, located more posteriorly [1, 4]. The contents of the interpeduncular cistern, namely the basilar artery, that can be frequently visualized, and its branches, the brainstem, the oculomotor nerves, and the dura mater of the clivus, should be considered [3, 4]. This endoscopic approach of the third ventricle is now considered the gold standard and one of the most frequently performed endoscopic surgeries [5].

Due to the complex anatomy of the third ventricle and the numerous relations with different important structures, complications of ETV have been described [6]. Bouras et al. reported an overall complication rate of 8.8% [6]. These complications ranged from periventricular neural structures trauma to intraoperative hemorrhage, causing permanent morbidity in 2.1% of cases [6], and were related to endoscope manipulation or perforation of the third ventricle floor [4]. These complications may include rupture of the basilar artery resulting in severe intraventricular hemorrhage and injury of nearby structures such as the thalamus, midbrain, or fornix [3, 4]. Also, neurocognitive complications are less frequent, but memory impairment, cognitive dysfunction, declined executive functions, and confusion were reported [1, 7] and related to damage to the fornix, mammillary bodies, anterior thalamus, and hippocampal formation [8].

Since Lespinasse used a urethroscope to reach the lateral ventricles in 1910, much has been done toward safer and more effective techniques [9]. Neuronavigation systems with preoperative computed tomography/magnetic resonance imaging (CT/MRI) appeared at the beginning of the twenty-first century and soon provided better spatial orientation, planning, and execution [10–13]. When visualizing the previously marked basilar artery, the neuronavigation-guided perforation of the ventricular floor was advantageous in patients with distorted anatomy [12]. Also, an MRI-guided protocol and endoscopic surgery with image guidance were found to reduce complications compared to the conventional blind procedure [4]. Moreover, a discrepancy between the external bony markings and internal cerebral anatomy was described [1]. This incongruity could translate into unnecessary traction and pressure on the cerebrum and limbic structures, including the fornix, resulting in cognitive deficits [1, 7, 8]. Therefore, the application of a stereotactic approach is advocated when choosing the trajectory from the burr hole to the third ventricle [1], minimizing the intraoperative endoscope manipulation, determining the distance from the basilar artery, and diminishing the procedure time, anesthetic and infection risk [11]. Studying the anatomy of periventricular structures is also acknowledged as essential to the execution of ETV [1]. The size of the IVF, the location of the infundibular recess and third ventricle floor, the termination of the basilar artery and the emergence of the oculomotor nerve are critical to obtain an optimized ETV trajectory [1]. Therefore, neuroanatomical studies to obtain normative morphometric data are one of the basic means to minimize complication rates [5, 14, 15].

Following a previous study that detailed the anatomic features of the third ventricle and, particularly, its floor [5], we now aim to clarify the anatomy of this ventricular cavity through the analysis of the stereotactic location of important neural and vascular structures in brain specimens of adult body donors and MRIs of adult individuals without cranioencephalic pathology. MRIs of adult non-obstructive hydrocephalic patients were also studied with the purpose of comparison and allowing better preoperative planning.

## Material and methods

### Brains collection

Fourteen brains were obtained from cadaver donation to the Unit of Anatomy of the Biomedicine Department of the Faculty of Medicine of the University of Porto. The donation for educational purposes was made in accordance with the Portuguese Act 274/99. This cadaveric brain study was approved by the Ethical Committee of the Centro Hospitalar Universitário São João/Faculty of Medicine of the University of Porto (no. 406/21).

The brains were extracted, embedded in a 10% formalin aqueous solution for several months, and preserved until their use, as described in detail elsewhere [16]. To avoid result errors, all brains were selected considering non-neurologic and psychiatric diseases. Before utilization, the brains were removed from the fixing solution and washed for 24 h in fresh cold running water to remove all presence of formalin.

**Fig. 1:**
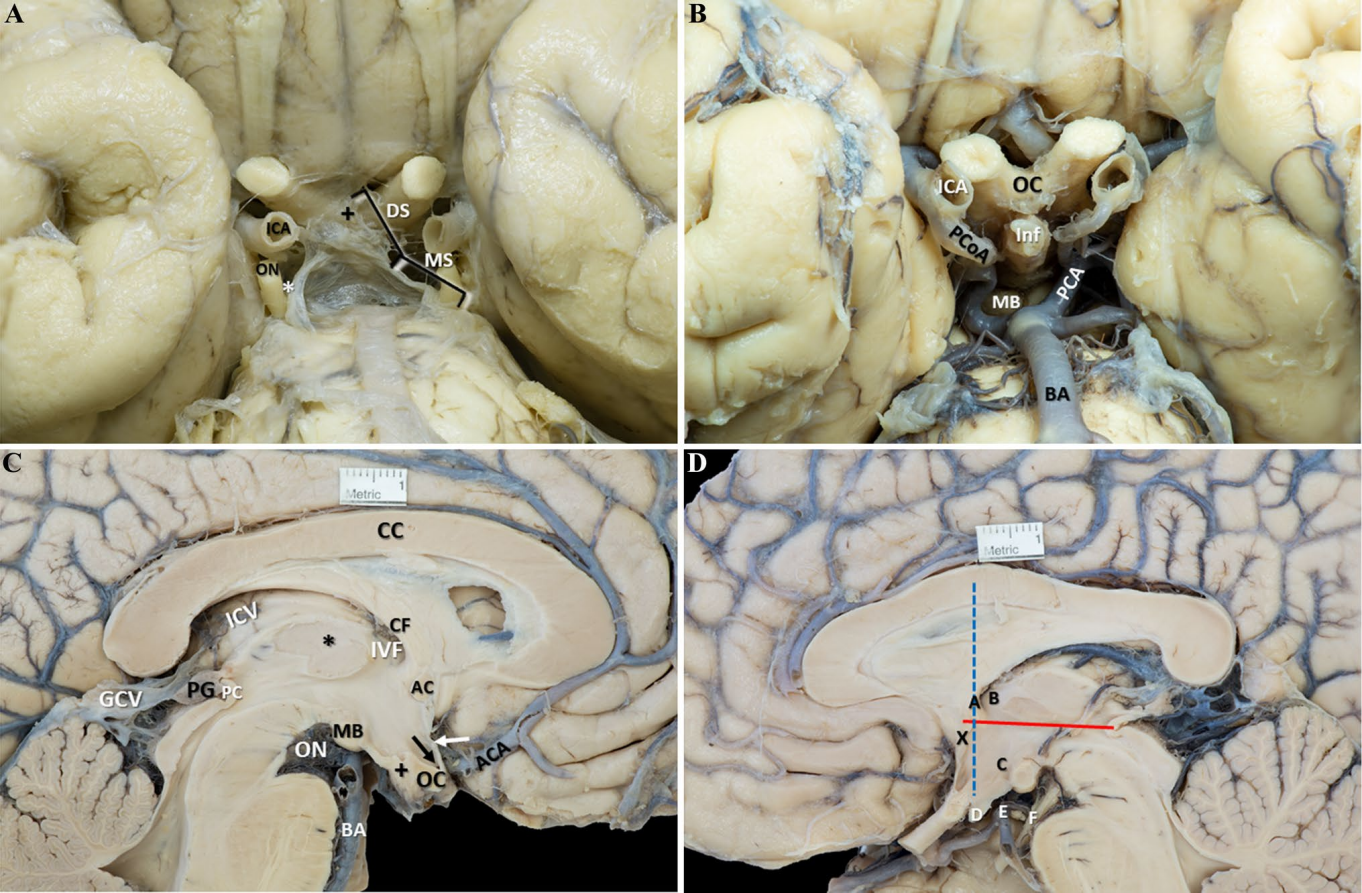
**A** External view of the interpeduncular fossa. The Liliequist membrane extends from the optic chiasm ( +) to the anterior pontine surface. Both diencephalic and mesencephalic segments of the Liliequist membrane and the pair of diencephalic-mesencephalic leaves (DML) (white arrow) are present. The DML gave arachnoid sheets to the anteromedial surface of the oculomotor nerve (*). **B** External view of the third ventricle floor in the interpeduncular fossa and its surrounding structures after Liliequist membrane dissection. **C** Medial view of the left cerebral hemisphere. The corpus callosum is seen cut in sagittal view and branches of the anterior cerebral artery follow its surface. The midline pineal gland projects posteriorly into the quadrigeminal cistern and is located just rostral to the superior colliculus. The great cerebral vein receives blood from deep structures via the internal cerebral vein. The third ventricle can be seen, including the interthalamic adhesion (*). The basilar artery terminates by giving off the posterior cerebral arteries within the interpeduncular cistern. The lamina terminalis (white arrow) stretches upward to fill the interval between the optic chiasm and the rostrum of the corpus callosum. The rostral portion of the third ventricle contains the supraoptic recess (black arrow) cranial to the optic chiasm. The tuber cinereum ( +) and mammillary body are located on the inferior surface of the hypothalamus. **D** Medial view of the right cerebral hemisphere. Several anatomic landmarks were used as reference points to better characterize the anatomy of the third ventricle. The anterior commissure-posterior commissure line was drawn (red line) and chosen as *Y*-axis as it serves as an accurate guide for identifying intracerebral structures. The *Z*-axis (blue dotted line) was drawn, passing through the posterior and superior portions of the anterior commissure. The intersection of both axes defined the stereotactic reference point (X). Six additional anatomic points (A–F) were used in the characterization of the anatomy of the third ventricle. **A**)Midline projection of the anterior border of the interventricular foramen; **B** midline projection of the posterior border of the interventricular foramen; **C** mammillary bodies anterosuperior portion; **D** most inferior point of the infundibular recess; **E** most superior point of the basilar artery; **F** midline projection of the emergence of the oculomotor nerve. *AC* anterior commissure; *ACA* anterior cerebral artery; *BA* basilar artery; *CC* corpus callosum; *CF* Column of the fornix; *DS* diencephalic segment; *GCV* great cerebral vein; *ICA* internal carotid artery; *ICV* internal cerebral vein; *Inf* infundibulum; *IVF* interventricular foramen; *MB* mammillary body; *MS* mesencephalic segment; *OC* optic chiasm; *ON* oculomotor nerve; *PC* posterior commissure; *PCA* posterior cerebral artery; *PCoA* posterior communicating artery; *PG* pineal gland (colour figure online)

The interpeduncular fossa of the brain specimens was observed (Fig. 1A) If the arachnoid, and particularly the arachnoid leaflet known as the Liliequist membrane, were present, they were removed to allow the visualization of the deep-located structures of the interpeduncular fossa (Fig. 1B). The optic chiasm, the pituitary stalk, the mammillary bodies, the basilar artery and branches, and the oculomotor nerve were identified after the dissection. Sometimes the oculomotor nerve was not present, but the sulcus, where it emerges in the medial surface of the midbrain, was visible.

Subsequently, brains were divided in the midsagittal plane, and their medial surfaces were examined (Fig. 1C and D). The superior convex margin of the third ventricle extends from the IVF to the suprapineal recess. The posterior wall of the third ventricle, from superior to inferior, includes the habenular commissure, the pineal gland, the pineal recess, and the posterior commissure. The rostral portion of the third ventricle contains the supraoptic recess, cranial to the optic chiasm, and the infundibular recess, caudal to the optic chiasm. The anterior commissure and lamina terminalis extend along the anterior third ventricle from the IVF to the supraoptic recess. The anterior part of the floor of the third ventricle consists of the supraoptic and infundibular recesses, and the tuber cinereum. There is an intermediate portion that spans from the mammillary bodies to the posterior aspect of the interpeduncular space. Finally, the posterior part of the floor is situated just above the cerebral peduncles extending to the cerebral aqueduct. The lateral walls of the third ventricle are delimited cranially by the thalami and inferiorly by the hypothalamus and subthalamus. An inconsistent interthalamic adhesion connecting the two thalami is seen (Fig. 1C and D).

**Fig. 2:**
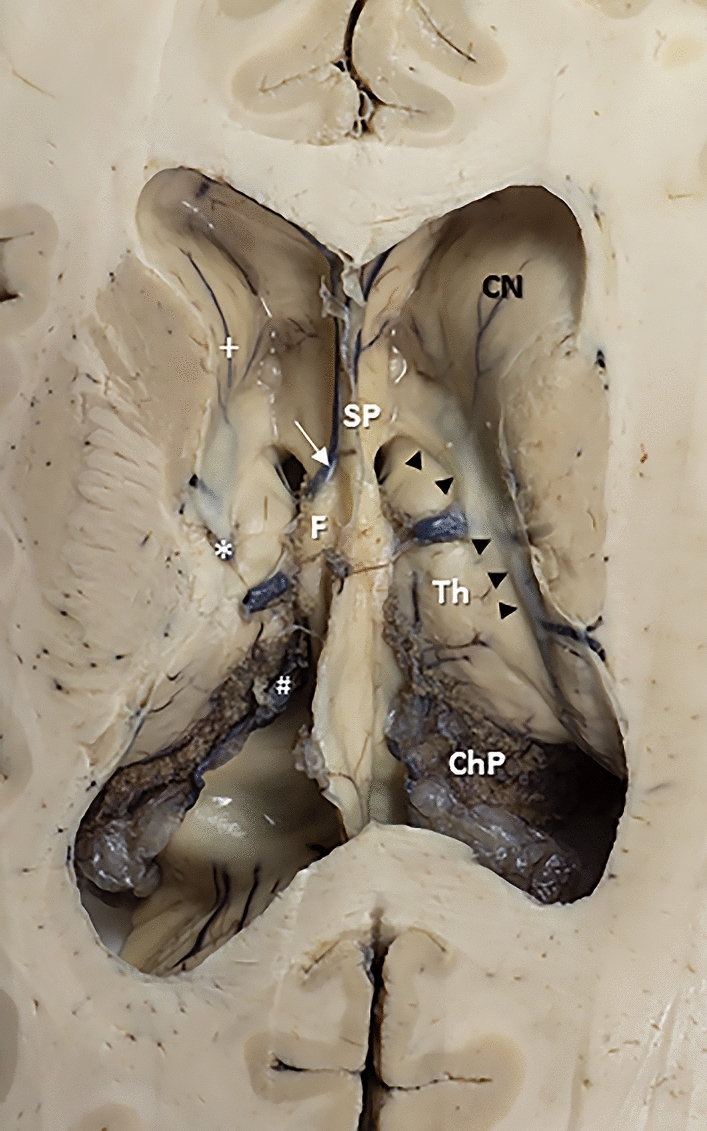
Superior view of the floor of the frontal horns and bodies of the lateral ventricles to understand and document the trajectory followed by an endoscope in an approach to the third ventricle. In the lateral walls of both ventricles structures it is seen the head of the caudate nucleus. In each ventricle, the thalamostriate vein (*), caudate veins ( +), choroidal veins (#), and septal veins (white arrow) coalesce and drain through the posterior border of the interventricular foramen (§). The body of the fornix project from the inferior edge of the septum pellucidum. The column of the fornix passes anterior to the interventricular foramen (black arrow). The stria terminalis covers the thalamostriate vein in the caudothalamic groove between the medial edge of the caudate nucleus and the superior surface of the thalamus, marking a line of separation between both nuclei (arrowheads). The choroidal plexus lies on the choroidal fissure. *ChP* choroidal plexus; *CN* caudate nucleus (head); *F* fornix; *SP* septum pelucidum; *Th* thalamus (colour figure online)

From the total of brain hemi-sections obtained, we selected 23 brain hemi-sections that exhibited preserved integrity of the largest number of anatomic landmarks relevant to the morphometric analysis. An additional brain was sectioned in a horizontal plane to understand and document the trajectory followed by an endoscope (Fig. 2).

### Imaging collection

Photographs were taken to demonstrate the anatomic architecture of the medial surfaces of the selected brain hemi-sections using a Nikon D750 SLR with a 105 mm Micro Nikkor lens mounted in a tripod at a constant distance from the brains. Images were captured along with a metric scale ruler placed perpendicularly to the lens. All the digital photographs included in this study were edited for white balance, contrast, sharpness, and cleaning of dust in the sensor using Photoshop CC (Adobe, CA).

### Morphometric measurements in brain hemi-sections

Several anatomical landmarks were used as reference points to better characterize the anatomy of the third ventricle, namely the anterior commissure (AC), the posterior commissure (PC), the IVF, the mammillary bodies (MB), the infundibular recess (IF), the basilar artery (BA) and the emergence of the oculomotor nerve (ON). Because the present study was both of anatomic and neuroradiological importance, the anterior commissure-posterior commissure (AC-PC) line was chosen as an important landmark, as it serves as an accurate guide for the identification of intracerebral structures. All morphometric parameters described were analyzed using the public domain software ImageJ (Fiji 1.46, National Institutes of Health, Bethesda, Maryland, USA), which allowed the establishment of distances and the identification and analysis of the defined stereotactic reference points.

First, after identifying the location of both anterior and posterior commissures in the medial cerebral hemispheric surface, we defined the AC-PC line (Fig. 1D, red line) as the *Y*-axis. The *Z*-axis (Fig. 1D, blue dotted line) was defined as being perpendicular to the *Y*-axis and passing through the posterior and superior portions of the AC in an upward direction. The intersection of both axes was defined as our stereotactic reference point with the coordinates (*X*, *Y*, *Z*) = (0, 0, 0). Furthermore, six other points were defined as follows:

A. IVF_A: midline projection of the anterior border of the interventricular foramen.
B. IVF_P: midline projection of the posterior border of the interventricular foramen.
C. MB: mammillary bodies anterosuperior portion.
D. IF: most inferior point of the infundibular recess.
E. BA: most superior point of the basilar artery or its midline projection when lateralized.
F. ON: midline projection of the emergence of the oculomotor nerve.

For the specimens in which the oculomotor nerve was not clearly identified, we estimated its location on the medial aspect of the crus cerebri in the interpeduncular fossa. All stereotactic reference coordinates (points A–F) were measured in millimeters using ImageJ software and determined independently by two investigators.

### Brain MRIs collection

Since MRI techniques minimize distortion and constitute a detailed anatomically and accurate element for imaging studies, we retrospectively reviewed 3D T1-weighted post-contrast sequences (3D SPGR T1) of 224 brain MRIs from the archive of the Radiology Service of the Portuguese Institute of Oncology of Porto, Porto, Portugal. Of the selected scans, 200 referred to adult (≥ 18 years old) subjects with normal brain morphology, i.e., without intracranial space-occupying lesions, cerebral edema, or hydrocephalus. The remaining 24 scans referred to adult (≥ 18 years old) subjects with non-obstructive hydrocephalus. Inclusion criteria for hydrocephalus include a bicaudate index larger than 95th percentile for age, effacement of pericerebral spaces and stretching of corpus callosum [17]. The MRIs use was approved by the institutional review boards of the Portuguese Institute of Oncology of Porto, Porto, Portugal, and in compliance with the Helsinki Declaration.

### Morphometric measurements in brain MRIs

**Fig. 3:**
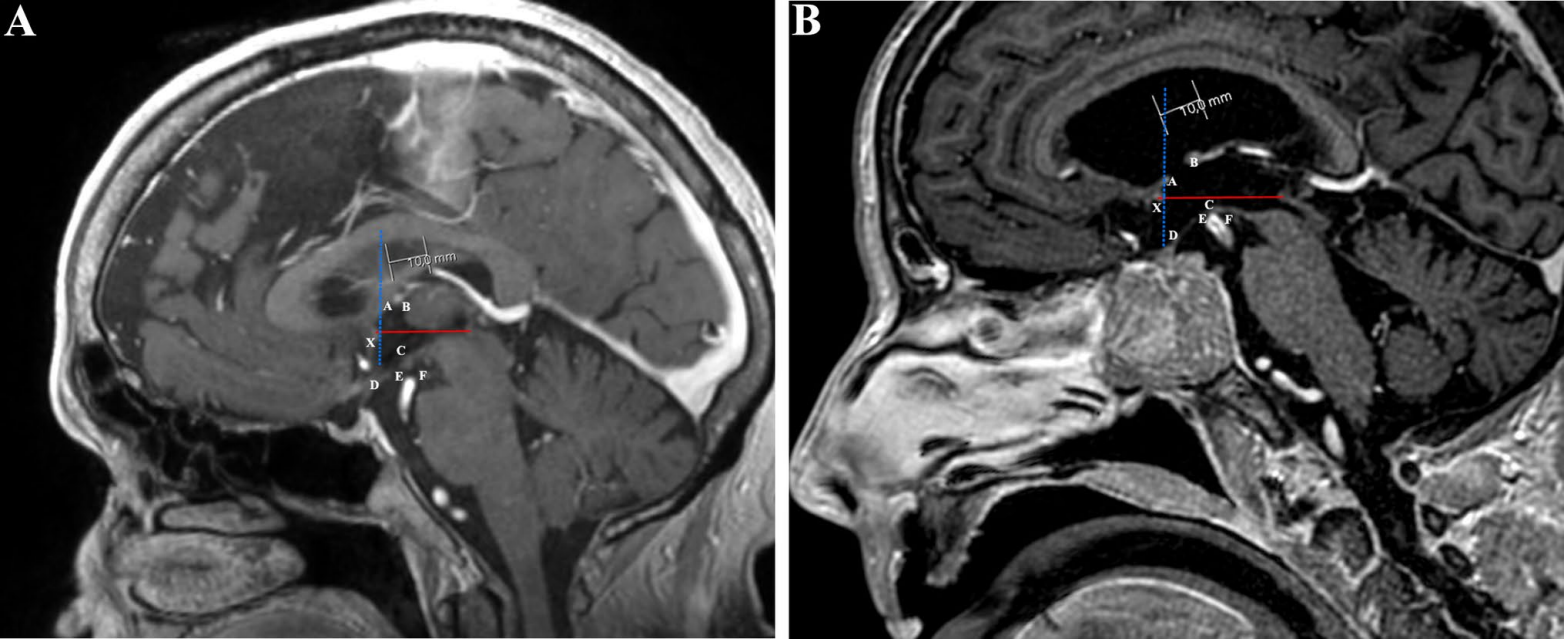
**A** Brain MRI. 3D T1-weighted post-contrast, midsagittal plane from normal subjects. The same anatomic landmarks were used as reference points. The anterior commissure-posterior commissure line was drawn (red line) and chosen as *Y*-axis. The *Z*-axis (blue dotted line) was drawn passing through the posterior and superior portions of the anterior commissure. The intersection of both axes defined the stereotactic reference point (X). Six additional anatomic points (A–F) were used in the characterization of the anatomy of the third ventricle. **B** Brain MRI. 3D T1-weighted post-contrast, midsagital plane from subjects with hydrocephalus. The same anatomical landmarks were used as reference points. A—Midline projection of the anterior border of the interventricular foramen; B—Midline projection of the posterior border of the interventricular foramen; C—Mammillary bodies anterosuperior portion; D—Most inferior point of the infundibular recess; E—Most superior point of the basilar artery; F—Midline projection of the emergence of the oculomotor nerve. Scale bar 10 mm (colour figure online)

In the midsagittal plane, a coordinate plane was similarly defined as described for the morphometric measurements in the brain hemi-sections. The same stereotactic reference points were defined (Fig. 3A, B), and their coordinates were measured in millimeters. Concerning the midline projection of the interventricular foramina and oculomotor nerve, we performed different measurements for both the right and left sides. All measurements were recorded by three investigators.

### Statistical analysis

An initial descriptive analysis of the six anatomic landmarks of the cadaver and MRI brain hemi-sections was performed. The coordinates of the healthy brain MRIs were normally distributed and are so presented as means and standard deviations; on the other hand, the coordinates of the cadaver and hydrocephalus brains had a more asymmetric distribution, and medians and 25th/75th percentiles were deemed more appropriate to describe them.

Subsequently, possible gender and laterality differences were explored in the MRI healthy and hydrocephalus brains through t tests and Mann–Whitney-*U* tests, respectively. Finally, the anatomic landmarks’ measurements of the normal MRI brains were compared with the same coordinates in the cadaver and the hydrocephalus brains, again using Mann–Whitney-*U* tests, considering the aforementioned distributions of the variables. The significance level was set at *α* = 0.01. Statistical analysis was conducted using SPSS statistical software package version 26 (SPSS inc., Chicago, IL, USA).

## Results

### Qualitative observation of the formalin-fixed brain hemi-sections

In the horizontal sections, the limits of the lateral ventricles were recognized (Fig. 2). The frontal horns of the lateral ventricle, typically presenting no choroid plexus, were visualized. The caudate nucleus forming the lateral wall of the lateral ventricle and the septum pellucidum forming the medial wall were observed on each side. The choroid plexus on the floor and the IVF characterizing the central part of the lateral ventricle were conspicuously present. The thalamostriate vein (laterally) and the septal veins (medially) were identified. The two IVFs could be observed leading to the third ventricle. The fornix and the relation with the thalamus, the columns of the fornix (anterior limit of the IVF), and the anterior tubercle of the thalamus (posterior limit of the IVF) were visualized. The stria terminalis was also identified between the thalamus and the body of the caudate nuclei. More posteriorly, the choroidal glomus was observed in the collateral trigone of each lateral ventricle.

### Morphometric measurements in formalin-fixed brain hemi-sections

Coordinates of the stereotactic reference points were measured in 12 right, and 11 left brain hemi-sections and are presented in Table 1. Due to the specimens’ anatomic limitations, we were unable to identify the basilar artery in two brain hemi-sections and the oculomotor nerve in four of the brain hemi-sections. From the remaining specimens, seven oculomotor nerves were not clearly visualized, and therefore the location was estimated as previously described.

**Table 1:** Stereotactic coordinates of the anatomical landmarks measured in human brain hemi-sections, normal brain MRIs and hydrocephalus brain MRIs (at level *X* = 0)

Point	Coordinate	Brain hemi-sections				Normal brain MRIs			Hydrocephalus brain MRIs			
		*n*	Median	P25	P75	*n*	Mean ± SD	95% CI	*n*	Median	P25	P75
IVF_A	Y	23	– 2.10	– 2.40	– 1.60	400	– 1.38 ± 1.278	– 1.50; – 1.25	48	– 2.20	– 2.70	– 1.48
	Z		4.10	3.50	4.60		4.84 ± 1.629	4.68; 5.00		2.80	2.20	3.48
IVF_P	Y	23	– 3.20	– 3.30	– 2.60	400	– 5.15 ± 1.861	– 5.33; – 4.96	48	– 7.80	– 9.40	– 6.93
	Z		4.70	3.70	5.20		6.73 ± 1.697	6.56; 6.90		6.45	6.10	7.13
MB	Y	12	– 9.50	– 11.28	– 9.00	200	– 8.56 ± 1.184	– 8.72; – 8.39	24	– 11.30	– 14.25	– 10.10
	Z		– 8.40	– 9.15	– 7.10		– 7.01 ± 1.648	– 7.24; – 6.78		– 7.05	– 8.10	– 5.08
IF	Y	12	– 3.90	– 5.83	– 2.58	200	– 0.49 ± 1.597	– 0.72; – 0.27	21	– 1.60	– 2.20	– 0.80
	Z		– 12.10	– 13.65	– 10.40		– 13.34 ± 1.781	– 13.65; – 13.15		– 14.20	– 14.95	– 12.50
BA	Y	10	– 12.05	– 16.28	– 9.70	200	– 9.79 ± 2.218	– 10.10; – 9.48	24	– 12.15	– 14.23	– 11.53
	Z		– 21.15	– 23.20	– 17.30		– 13.97 ± 4.083	– 14.54; – 13.40		– 14.15	– 16.30	– 11.43
ON	Y	19	– 14.00	– 16.10	– 13.50	400	– 14.15 ± 1.853	– 14.33; – 13.97	48	– 16.45	– 18.98	– 14.18
	Z		– 12.10	– 13.50	– 10.90		– 11.22 ± 1.687	– 11.38; – 11.05		– 10.45	– 11.30	– 9.75

BA: most superior point of the basilar artery or its midline projection when it was lateralized; IF: most inferior point of the infundibular recess; IVF_A: midline projection of the anterior border of the interventricular foramen; IVF_P: midline projection of the posterior border of the interventricular foramen; MB: mammillary bodies anterosuperior portion; ON: midline projection of the emergence of the oculomotor nerve; P25: percentile 25; P75: percentile 75. Measurements are presented in millimeters

### Morphometric measurements in brain MRIs

Coordinates of the stereotactic reference points were measured in 400 hemispheres from 200 brain MRIs from healthy subjects. From the total number of brain MRIs analyzed, 166 (41.5%) corresponded to male subjects and 234 (58.5%) from female subjects. The mean age of subjects studied was 59.8 years (standard deviation (SD) = 13.54). Measurements are presented in Table 1.

Gender differences in stereotactic coordinates were found when comparing data from brain MRIs according to sex. Data was presented as mean ± SD for each of the stereotactic anatomic landmarks studied in Table 2. Particularly, IVF_A point was more superiorly and ON point more inferiorly located in female subjects when compared to male subjects (*p* = 0.007 and *p* = 0.009, respectively). On the other hand, IVF_P, MB, BA, and ON points were more anteriorly located in female subjects when compared to male subjects (*p* = 0.003, *p* < 0.001, *p* = 0.001, and *p* < 0.001, respectively).

**Table 2:** Comparative analysis of the measurements performed in brain MRIs from male and female subjects

Point	Coordinate	Sex	Mean ± SD	Mean difference (95% CI)	*p* value
IVF_A	Y	Male	– 1.53 ± 1.321	– 0.269 (– 0.526; – 0.012)	0.040
		Female	– 1.26 ± – 1.237		
	Z	Male	4.58 ± 1.588	– 0.441 (– 0.762; – 0.120)	**0.007**
		Female	5.02 ± 1.637		
IVF_P	Y	Male	– 5.47 ± 1.830	– 0.550 (– 0.917; – 0.182)	**0.003**
		Female	– 4.92 ± 1.853		
	Z	Male	6.69 ± 1.406	– 0.069 (– 0.392; 0.254)	0.675
		Female	6.76 ± 1.879		
MB	Y	Male	– 9.05 ± 1.186	– 0.850 (– 1.171; – 0.529)	** < 0.001**
		Female	– 8.20 ± 1.054		
	Z	Male	– 6.91 ± 1.957	0.164 (– 0.332; 0.659)	0.514
		Female	– 7.08 ± 1.392		
IF	Y	Male	– 0.71 ± 1.617	– 0.373 (– 0.826; 0.079)	0.105
		Female	– 0.34 ± 1.571		
	Z	Male	– 13.51 ± 1.840	– 0.183 (– 0.692; 0.327)	0.480
		Female	– 13.32 ± 1.741		
BA	Y	Male	– 10.41 ± 2.306	– 1.074 (– 1.699; – 0.450)	**0.001**
		Female	– 9.34 ± 2.049		
	Z	Male	– 13.75 ± 4.580	0.378 (– 0.823; 1.580)	0.535
		Female	– 14.12 ± 3.703		
ON	Y	Male	– 15.04 ± 1.737	– 1.513 (– 1.855; – 1.172)	** < 0.001**
		Female	– 13.52 ± 1.669		
	Z	Male	– 10.94 ± 1.961	0.470 (0.118; 0.822)	**0.009**
		Female	– 11.41 ± 1.435		

IVF_A: midline projection of the anterior border of the interventricular foramen; IVF_P: midline projection of the posterior border of the interventricular foramen; MB: mammillary bodies anterosuperior portion; IF: most inferior point of the infundibular recess; BA: most superior point of the basilar artery or its midline projection when it was lateralized; ON: midline projection of the emergence of the oculomotor nerve. Measurements are presented in millimeters. Statistically significant *p* values in bold

In addition, regarding the stereotactic anatomic points whose midline projection was considered, only IVF_A and IVF_P points’ Y coordinates were statistically significant when comparing differences between left and right hemispheres (Table 3). Both IVF_A and IVF_P were located more posteriorly in the left hemispheres (*p* < 0.001 in both).

**Table 3:** Comparative analysis of the measurements performed in left and right hemispheres in brain MRIs

Point	Coordinate	Side	Mean ± SD	Mean difference (95% CI)	*p* value
IVF_A	Y	Right	− 1.08 ± 1.296	0.592 (0.347; 0.836)	** < 0.001**
		Left	− 1.67 ± 1.190		
IVF_P	Y	Right	− 4.67 ± 1.852	0.948 (0.593; 1.302)	** < 0.001**
		Left	− 5.62 ± 1.751		

IVF_A: midline projection of the anterior border of the interventricular foramen; IVF_P: midline projection of the posterior border of the interventricular foramen. Measurements are presented in millimeters. Statistically significant *p* values in bold

### Morphometric measurements in hydrocephalus brain MRIs

A total of 24 brain MRIs from subjects with hydrocephalus were analyzed, and data concerning the same anatomic stereotactic coordinates analysis were presented in Table 1. From those 48 hemispheres, 22 (45.8%) were from male subjects, and 26 (54.2%) were from female subjects. The median of ages from subjects studied was 65.0 years (P25 = 38.50; P75 = 74.75). During data recordings, we were unable to identify the infundibular recess in three hemispheres due to image distortion and inappropriate MRI slice thickness.

Concerning gender and cerebral asymmetries, no statistically significant differences were found in brain MRI stereotactic coordinates from subjects presenting hydrocephalus.

### Comparative morphometric measurements between formalin-fixed brain hemi-sections and brain MRIs

Data regarding differences in morphometric measurements between formalin-fixed brain hemi-sections and brain MRIs were presented in Table 4. Data related to morphometric measurements in formalin-fixed brain hemi-sections showed that IVF_A, MB, IF, and BA points were located more posteriorly (*p* = 0.008, *p* = 0.002, *p* < 0.001 and *p* = 0.003, respectively) and IVF_P was more anteriorly located (*p* < 0.001) when compared to morphometric measurements from healthy brain MRIs. In addition, IVF_P and BA points were also located in a more inferior position compared to data from healthy brain MRIs (*p* < 0.001).

**Table 4:** Comparative analysis of the measurements performed in brain hemi-sections and in brain MRIs from healthy subjects

Point	Coordinate	Group	Median	Difference	*p* value
IVF_A	Y	Cadaver	– 2.10	– 0.70	**0.008**
		Normal MRI	– 1.40		
	Z	Cadaver	4.10	– 0.60	0.010
		Normal MRI	4.70		
IVF_P	Y	Cadaver	– 3.20	2.00	** < 0.001**
		Normal MRI	– 5.20		
	Z	Cadaver	4.70	– 1.95	** < 0.001**
		Normal MRI	6.65		
MB	Y	Cadaver	– 9.50	– 1.00	**0.002**
		Normal MRI	– 8.50		
	Z	Cadaver	– 8.40	– 1.30	0.010
		Normal MRI	– 7.10		
IF	Y	Cadaver	– 3.90	– 3.20	** < 0.001**
		Normal MRI	– 0.70		
	Z	Cadaver	– 12.10	1.40	0.049
		Normal MRI	– 13.50		
BA	Y	Cadaver	– 12.05	– 2.20	**0.003**
		Normal MRI	– 9.85		
	Z	Cadaver	– 21.15	– 7.60	** < 0.001**
		Normal MRI	– 13.55		
ON	Y	Cadaver	– 14.00	0.10	0.421
		Normal MRI	– 14.10		
	Z	Cadaver	– 12.10	– 1.00	0.044
		Normal MRI	– 11.10		

BA: most superior point of the basilar artery or its midline projection when it was lateralized; IF: most inferior point of the infundibular recess; IVF_A: midline projection of the anterior border of the interventricular foramen; IVF_P: midline projection of the posterior border of the interventricular foramen; MB: mammillary bodies anterosuperior portion; ON: midline projection of the emergence of the oculomotor nerve. Measurements are presented in millimeters. Statistically significant *p* values in bold

### Comparative morphometric measurements between healthy subjects and hydrocephalus patients MRIs

No age differences were found between healthy subjects and hydrocephalus patients (*p* = 0.266). Differences according to stereotactic coordinate measurements are presented in Table 5. Taking into consideration data from hydrocephalus patients MRIs, all stereotactic points studied were localized more posteriorly compared to data from brain MRIs from healthy subjects, and differences were statistically significant. Furthermore, the IVF_A point was located more inferiorly, and ON point was more superiorly located when compared with brain MRIs from healthy subjects (*p* < 0.001 and *p* = 0.004, respectively).

**Table 5:** Comparative analysis of the measurements performed in brain MRIs from healthy and hydrocephalus subjects

Point	Coordinate	MRI group	Median	Difference	*p* value
IVF_A	Y	Normal	− 1.40	0.80	** < 0.001**
		Hydrocephalus	− 2.20		
	Z	Normal	4.70	1.90	** < 0.001**
		Hydrocephalus	2.80		
IVF_P	Y	Normal	− 5.20	2.60	** < 0.001**
		Hydrocephalus	− 7.80		
	Z	Normal	6.65	0.20	0.351
		Hydrocephalus	6.45		
MB	Y	Normal	− 8.50	2.80	** < 0.001**
		Hydrocephalus	− 11.30		
	Z	Normal	− 7.10	− 0.05	0.467
		Hydrocephalus	− 7.05		
IF	Y	Normal	− 0.70	0.90	**0.001**
		Hydrocephalus	− 1.60		
	Z	Normal	− 13.50	0.70	0.140
		Hydrocephalus	− 14.20		
BA	Y	Normal	− 9.85	2.30	** < 0.001**
		Hydrocephalus	− 12.15		
	Z	Normal	− 13.55	0.60	0.864
		Hydrocephalus	− 14.15		
ON	Y	Normal	− 14.10	2.35	** < 0.001**
		Hydrocephalus	− 16.45		
	Z	Normal	− 11.10	− 0.65	**0.004**
		Hydrocephalus	− 10.45		

BA: most superior point of the basilar artery or its midline projection when it was lateralized; IF: most inferior point of the infundibular recess; IVF_A: midline projection of the anterior border of the interventricular foramen; IVF_P: midline projection of the posterior border of the interventricular foramen; MB: mammillary bodies anterosuperior portion; ON: midline projection of the emergence of the oculomotor nerve. Measurements are presented in millimeters. Statistically significant *p* values in bold

## Discussion

The third ventricle is considered one of the most profound and inaccessible regions of the brain [5, 8, 18, 19]. During ETV, the anterior wall of the third ventricle near the lamina terminalis should be avoided due to the presence of the optic chiasm and the infundibular recess [2]. The columns of the fornix should not be manipulated to prevent neurocognitive alteration [8, 18]. Also, lesion of the hypothalamus or its vessels can result in endocrine and metabolic disturbances [8, 18]. The fenestration of the floor of the third ventricle should be done posterior to the infundibular recess and anterior to the basilar artery and mammillary bodies [5]. Not always visible through the semi-translucid floor of the third ventricle, the basilar artery must not be injured due to the risk of fatal blood loss [5, 6]. Therefore, to access the third ventricle endoscopically, profound knowledge of anatomy is imperative to avoid lesions of these critical structures [5, 8, 19].

Many studies have been reinforcing the association of ETV with neuronavigation systems to increase efficacy and minimize possible complications [1, 4, 11]. For example, using neuronavigation leads to less manipulation and displacement of the fornix and hypothalamus compared to manually planned trajectories [7]. The better characterization of the anatomy of the third ventricle is also instrumental.

Therefore, in this study, we obtained stereotactic coordinate measurements of several periventricular structures in cadaveric donor brains and MRIs of adult individuals without hydrocephaly that neurosurgeons could use when planning the surgical approach to ETV. Previously we used 26 adult cadaveric brains and 200 adult brain MRIs describing the third ventricle floor and related structures and concluded that, in older subjects and when the basilar artery caliber was larger, its apex was closer to the floor of the third ventricle [5]. Mavridis and Anagnostopoulou described the stereotactic localization of the anterior-inferior border of the IVF and the midpoint between it and the anterior commissure, using 44 cadaveric hemispheres to define a safer approach to the third ventricle [15]. Using the posterosuperior border of the anterior commissure as the stereotactic reference point, these authors found that the mean stereotactic *Y* coordinate of the IVF anterior-inferior border was -0.61 ± 1.58 mm and the mean stereotactic *Z* coordinate for the same point was 2.41 ± 2.93 mm [15, 20]. Comparing these measurements with the present results (mean ± SD: *Y* = − 1.92 ± 0.779 mm; *Z* = 3.94 ± 1.175 mm), it was found that the anterior border of the IVF seems to be placed slightly more posteriorly and superiorly, but the difference does not appear to be significantly different. This minor difference may be related to the different definitions chosen to point to the structure and the different methodologies used in the quantification. To compare with our results, we had to use the mean coordinates, although we believe that is not the best way to approach our results, considering the asymmetric distribution of these coordinates in the cadaveric brain specimens.

In the cadaveric brains, analysis regarding the differences between sides and sexes were not conducted due to an insufficient number of specimens and their anonymity. However, Mavridis and Anagnostopoulou found that the anterior border of the left IVF was located more posteriorly than the right one, even though there was no statistical significance [15].

Other morphometric data of the third ventricle were obtained from adult cadaveric hemispheres [18] with the intent to improve the subfrontal approach used to remove tumors and cyst formations, considering structures like the lamina terminalis, the genu of the corpus callosum, the anterior commissure, and the optic chiasm [18]. Other important structures were also aimed at by the same authors when taking into account the transcallosal-interforniceal approach [14]. Winkler et al. analyzed 72 MRI scans and morphometrically described structures involved in this approach: the IVF, the anterior commissure, the corpus callosum, and the columns of the fornix [21]. Morphometric descriptions of brain cadavers were also obtained from dissection with frameless stereotactic navigation [22] and three-dimensional reconstruction of the brains [23].

As the importance of associating data obtained from cadaveric dissection and MRIs has been highlighted [24, 25], this comparison was performed in the present study. We consider that these measurements obtained in normal MRIs contribute to the stereotactic description of the third ventricle due to the considerable number of MRIs analyzed. There are some statistically significant differences between genders in the stereotactic coordinates of some structures, but it is important to consider the magnitude of the differences found. In the present results, only two anatomic landmarks exceed 1 mm of difference in their median coordinates, which complies with the range of accepted errors in a neuronavigation system. We believe that differences of this extent do not constitute anatomic pitfalls when planning an approach to the third ventricle [10, 13] and, therefore, have no clinical significance. We found that the anterior border of the IVF was more superiorly located in females, whereas its posterior border and the anterosuperior portion of the mammillary bodies were more anteriorly located in females. The oculomotor nerve was more inferiorly located in females. The oculomotor nerve and basilar artery were also more anteriorly located in females. Relative to left and right-side asymmetries, the differences were even smaller and statistically significant only on the location of the IVF, which was placed more posteriorly in the left hemisphere.

Concerning the gender issue, it was previously reported that the height, anteroposterior diameter, and transverse diameter of the third ventricle were related to the individual anatomic variability of the skull shape and gender. Also, most published reports reviewed elsewhere reported that these morphologic parameters were greater in males [26]. Another study using other landmarks in MRI found no differences between genders except for the distance from the interforniceal insertion to the optic chiasm, which was greater in males [23].

The comparison between the brain specimens and MRIs measurements presents relatively minor differences. Although most of them are statistically significant, they may not be clinically important when using neuronavigation systems during ETV. In fact, most of the stereotactic coordinates were resilient to the fixation procedure, and most of the dimensions and distances were maintained. It is important to note that an error of 3.7 mm in image-guided neuronavigation systems during ETV was considered to be clinically acceptable [13]. There was a difference, barely greater than 3 mm, in the *Y* coordinate related to the inferior point of the infundibular recess (3.2 mm). A greater distance was observed in the *Z* coordinate of the most superior point of the basilar artery (7.6 mm). This distance can be justified due to post-mortem shrinkage of vascular structures, the absence of CSF in the cadaveric brain and its presence in the interpeduncular cistern in the MRIs. Note that the midline projection of the emergence oculomotor nerve, also present in the interpeduncular cistern, differs only by 1 mm.

It could be argued that the reduced number of brain specimens could justify the absence of major differences. However, they are in line with the absence of variations between fresh and fixed brains regarding ventricle dimensions, described by others [18]. The present quantifications corroborate old data stating that the ventricular system morphometry in fresh and fixed brains was similar. It was reported that formalin-fixed brains had only a decrease of 1.2% in length and 1.5% in width compared to fresh brain tissue, which is entirely insignificant, even negligible [18]. The cadaveric brains provide valuable information despite the differences between patients undergoing surgery due to the absence of blood flow and CSF, post-mortem changes in the vascular and neural structures, and changes due to formalin fixation. Also, the absence of ventricle collapse following fixation is another factor to be considered [27]. Note that in vivo endoscopic procedures provide a redefinition of the anatomy of ventricular structures. The CSF pressure may be increased in most types of hydrocephaly and induce changes in the volume of some structures [20, 28]. Our data contributes to the knowledge of the anatomy of the third ventricle and it is important for the clinical and surgical practice in general.

Interestingly, a strong correlation also exists between high-definition 7 T MRI and the Klingler white matter dissection technique in cadaveric brains and allied to neuronavigation procedures, which can increase the understanding of the structural connections of other complex areas of the brain [24]. We can conclude that despite all the changes following death and fixation procedures, cadaveric material remains a critical tool for training surgical skills and medical education [29]. Nowadays, an association of cadaveric material with, for example, cadaveric CT and MRI scans has been suggested as a way of adding together the immediate visualization of structures and its tridimensional understanding [29].

Although ETV is considered the first procedure adopted in obstructive hydrocephalus cases, and its role in non-obstructive hydrocephalus is obviously very limited and debated, we added MRIs from non-obstructive hydrocephalic patients to quantify the magnitude of the changes of the stereotactic coordinates.

Surprisingly, comparing the normal MRI brains with the MRIs from hydrocephalic adult patients revealed that the differences of all stereotactic coordinates were smaller than 3 mm. It was previously reported that the distance between the basilar artery’s apex and the third ventricle’s floor was significantly smaller in the brains of patients with ventricular dilation (2.75 mm versus 6.54 mm in the control patients) [27]. In our results, it was found that there was a significant difference of 2.3 mm in the Y stereotactic coordinate (− 9.85 mm in normal patients versus − 12.15 mm in hydrocephalic brains). However, no significant difference was found in the Z coordinate, differing from the comparisons made with cadaveric brains. This is consistent with the smaller distance to the third ventricle’s floor when ventricles are dilated due to hydrocephaly. This conditions may lead to a downward depression of the floor of the third ventricle in obstructive and non-obstructive hydrocephaly [27, 30], but not of the artery’s apex. Therefore, additional caution is required when perforating the floor of the third ventricle in hydrocephalic subjects to avoid injuring this vessel.

We can advance some reasons that could explain the absence of major differences between normal and hydrocephalus brain MRIs that theoretically should present substantial changes in the stereotactic coordinates herein analyzed. The first reason may be the relatively small number of hydrocephalus MRI specimens and the inadequate slice thickness, making it challenging to identify the points of interest. The second reason is related to the dimensions considered in the present estimation, as there were the anteroposterior and craniocaudal diameters. We did not evaluate possible changes in the transverse diameter because all the MRIs analyzed were on the sagittal plane to be compared with cadaver data. Finally, the magnitude of the disease in the analyzed specimens may also have influenced the measurements. However, the relative stability of the stereotactic coordinates of most of the hydrocephalus structures should be considered when planning the surgery.

We stress that the normative quantitative data of the third ventricle presented here are very important and valuable to the neurosurgeons planning the approach to the third ventricle. However, it must be remembered that due to the high-individual variability, the MRIs of each patient and the optimal trajectory to the third ventricle must be planned individually. Determining the best operative technique and the individualization of the intervention, a CT and MRI are indispensable for evaluating the ventricular size. Finally, it is essential to remember that the presence of anatomic variations must be identified as they influence the success rate of ETV [31] as they can result in the loss of the anatomic landmarks and lead to a more technically demanding surgical procedure. The incidence of variations was reported to be 36% in a study of 25 patients, and the success rate dropped 44% in ETV of patients presenting anatomic variations [32].

The present study has some limitations. The absence of age and sex data concerning the cadaveric brain specimens and the relatively small number is justifiable due to the difficulty of obtaining this type of material and the protection of donor data. The number of MRIs from hydrocephalic patients is also small but allows verifying that some structures present their stereotactic coordinates unaltered. The presence of the transverse dimension would have allowed a better anatomic characterization of the third ventricle and hydrocephalus, and it will be considered in a future study. Finally, the presented measurements of the III ventricle might not be comparable to those of obstructive hydrocephalus since our data was obtained from non-obstructive hydrocephalic patients and, generally, ETV is not commonly use in those situations.

## Conclusion

With the present work, we obtained the stereotactic coordinates of relevant structures in cadaveric brains necessary for the ETV procedure and compared these coordinates with those obtained in the MRI of patients without neurosurgical pathology and hydrocephalic individuals. We found only minor statistically significant differences, and most of them of very small magnitude and probably not clinically significant. These normative quantitative data of the stereotactic coordinates of the third ventricle are necessary to evaluate the changes in the anatomy due to hydrocephalus that may distort topography and landmarks. Although these data are helpful, neurosurgeons must be aware that due to the high-individual variability of the patients and the possibility of anatomic variants, the optimal trajectory to the third ventricle must be planned individually. Determining the best operative technique and the individualization of the intervention, a precise angiographic study, CT and MRI are indispensable for the evaluation of the ventricular size and surgical planning of ETV and preservation of neurophysiological function and structures. We conclude that research using cadaveric brains to train neurosurgeons is helpful for accurate and detailed knowledge of the normal brain and is fundamental for the neurosurgical treatment of the diseased brain in the twenty-first century.

## Data Availability

Not applicable.
